# A cost-consequence analysis of the children’s administration oxygenation strategies trial (COAST) in severe pneumonia

**DOI:** 10.1371/journal.pgph.0005654

**Published:** 2026-01-08

**Authors:** Orlagh U. Carroll, Richard Grieve, Sarah Kiguli, Peter Olupot-Olupot, Robert O. Opoka, Florence Alaroker, Abner Tagoola, Mainga Hamaluba, Eva Nabawanuka, Damalie Nalwanga, Tom N. Williams, Karen Thomas, David A. Harrison, Paul Mouncey, Elisa Giallongo, Kathy Rowan, Kathryn Maitland, Zia Sadique

**Affiliations:** 1 Department of Health Services Research and Policy, London School of Hygiene & Tropical Medicine, London United Kingdom; 2 School of Medicine, Makerere University and Mulago Hospital Kampala, Kampala, Uganda; 3 Faculty of Health Sciences, Mbale Campus and Mbale Regional Referral Hospital Mbale (POO, WO), Busitema University, Mbale, Uganda; 4 Jinja Regional Referral Hospital Jinja, Jinja, Uganda; 5 Soroti Regional Referral Hospital, Soroti, Uganda; 6 Kilifi County Hospital and Kenya Medical Research Institute (KEMRI) Wellcome Trust Research Programme, Kilifi, Kenya; 7 Department of Infectious Disease and Institute of Global Health and Innovation, Division of Medicine, Imperial College, London, United Kingdom; 8 Royal Marsden NHS Foundation Trust, London, United Kingdom; 9 Intensive Care National Audit & Research Centre (ICNARC), London, United Kingdom; PLOS: Public Library of Science, UNITED STATES OF AMERICA

## Abstract

Oxygen supplementation is a recommended treatment for children with severe pneumonia or hypoxaemia. The open, fractional-factorial Children’s Oxygen Administration Strategies Trial (COAST) recruited Kenyan and Ugandan children with severe pneumonia and hypoxaemia. Participants in the severe hypoxaemia stratum (SpO_2_ < 80%) were randomised to high-flow nasal therapy (HFNT) or low-flow oxygen (LFO), and in the hypoxaemia stratum (SpO_2_ 80–91%) to HFNT, LFO or permissive hypoxaemia (ratio 1:1:2). The trial stopped early and there is ongoing uncertainty about the clinical benefits of the alternative strategies. There is a lack of evidence about the relative costs, of alternative oxygen delivery for critically-ill children in low- and middle- income countries. We used data from COAST to conduct a cost-consequence analysis of the treatment strategies. We measured resource use for 28 days post-randomisation (n = 1,842). Resources included oxygen delivery, medications, blood and fluid products, diagnostic tests, point of care tests, hospital admission and length of stay. We calculated the total costs and reported the incremental costs as the difference in the mean total costs between groups, adjusting for baseline differences. In the severe hypoxaemia stratum, the mean total cost was $393.04 for HFNT and $218.73 for LFO. In the hypoxemia stratum, the mean total costs were $391.95 (HFNT), $198.26 (LFO) and $167.80 (permissive). The adjusted cost difference between HFNT versus LFO and liberal versus permissive was $184.43 (95% CI l: $127.90, $240.95), and $124.01 (95% CI: $99.53, $148.49), respectively. The differences of HFNT and LFO versus permissive were $216.22 (95% CI: $160.77, $271.68) and $31.80 (95% CI: $11.49, $52.11), respectively. For children with severe hypoxaemia, HFNT is more costly than LFO. For children with hypoxaemia, either of HFNT or LFO were more costly than permissive hypoxaemia. The main driver of costs for HFNT is the high cost of equipment and consumables; other costs were similar across treatment groups in both strata, as were health outcomes.

## 1. Introduction

Pneumonia is the leading cause of death in children under 5 years old worldwide, accounting for 0.75 million deaths—14% of all deaths in children under 5 years old [1]. Globally, there are over 1,400 cases of pneumonia per 100,000 children, with the greatest incidence occurring in low and middle income countries (LMICs) [2–4]. A large proportion of pneumonia episodes progress to severe pneumonia [5], leading to hospitalisation with supportive treatment and careful monitoring. The World Health Organization (WHO) recommends presumptive antibiotic treatment and oxygen for those with clinically defined severe pneumonia and/or hypoxaemia (peripheral oxygen saturation (SpO_2_) < 90% [3,6,7]).

Supplemental oxygen is recommended for children with severe pneumonia and hypoxaemia however, there are clinical and logistic/technical challenges in the delivery of oxygen in LMICs [6]. The targeted use of oxygen and simple, non-invasive methods of respiratory support may be a highly effective means of improving outcome, but there is a lack of robust clinical evidence from adequately powered randomised controlled trials (RCT) [8] about the optimal oxygen saturation threshold that results in benefit and the best strategy for delivering oxygen (refer to section 1.9 of [9]). Moreover, reliable supplies of medical oxygen are also limited in health care facilities in Africa [10]. There are two main sources of oxygen supply in hospitals: high pressured cylinders and oxygen concentrators. In general, high-pressure cylinders do not require electricity but they need to be refilled and transported which in LMICs can be costly and raise logistical challenges [11]. Oxygen concentrators are machines that concentrate oxygen at the bedside by absorbing nitrogen from atmospheric air. While concentrators can be a reliable and efficient source of oxygen, the capital costs of the concentrators are relatively higher, and they require regular maintenance and a reliable source of power, which in LMIC health facilities is often not available [11,12]. Although oxygen is well established as a treatment for hypoxemic pneumonia, the costs and consequences of alternative ways to address hypoxaemia have received little to no attention in current global health strategies.

In order to help address this gap in knowledge, the Children’s Oxygen Administration Strategies Trial (COAST) aimed to evaluate the clinical effectiveness of the following alternative strategies: whether (a) for children with severe pneumonia and severe hypoxaemia (SpO_2_ < 80%), respiratory support with high-flow nasal therapy (HFNT) versus low-flow oxygen (LFO) delivery (standard care) decreases mortality (at 48 hours and up to 28 days), and (b) for children with hypoxaemia (SpO_2_ 80–91%), liberal oxygenation strategies (HFNT or LFO) compared with a permissive hypoxaemia strategy decreases mortality (at 48 h and up to 28 days). This RCT enrolled Ugandan and Kenyan children aged between 28 days and 12 years old with respiratory distress complicated by hypoxaemia [9]. COAST was stopped early due to feasibility concerns from the Trial Steering Committee (further details available in Supplement 10b of [13]). The findings of the trial, albeit underpowered for the primary endpoint (mortality at 48 hours), found no significant difference in the clinical benefit of any of the oxygenation strategies including HFNT [13,14]. However, there is a lack of scientific data on the relative costs of the standard and alternative modes of oxygen delivery. An economic evaluation of the trial may provide data for future trial designs and, in the interim, granular information on the overall costs of treatment which is useful for decision-makers making choices on resource allocation. This paper aims to conduct a cost consequence analysis of these alternative routes of oxygen delivery strategies in critically-ill children using patient level data from COAST.

## 2. Methods

### 2.0. Ethics statement

The COAST protocol was approved by the School of Medicine Research Ethics Committee, Makerere University, Kampala, Uganda, Kenya Medical Research Institute Scientific Ethics and Review Unit, Nairobi, Kenya and Imperial College Research Ethics Committee approved the trial (ISRCTN15622505).

### 2.1. Overview

This economic evaluation takes the form of a cost-consequence analysis, which is a recommended form of evaluation in settings such as those evaluating interventions for children where there are challenges in reporting results according to a single endpoint (e.g., the disability adjusted life year), and there is interest in reporting the relative costs alongside a range of clinical outcome measures [15,16]. In describing the methods for the cost-consequence analysis from a hospital perspective, we describe the main elements of the RCT required for the assessment of resource use and costs, and refer the reader who requires further details to the published protocols and reports of the main clinical outcomes of COAST. Additional information regarding the ethical, cultural, and scientific considerations specific to inclusivity in global research is included in S2 Checklist.

### 2.2. Setting and selection

COAST was a two-stratum multicentre, open, fractional factorial RCT conducted in four Ugandan and two Kenyan hospitals. Children with previous diagnosed but uncorrected cyanotic heart disease, chronic lung disease (excluding asthma), children given oxygen at another health facility (or > 3 hours at the current hospital) or previous COAST enrolment were excluded. Children aged 28 days to 12 years, hospitalised with a history of respiratory illness and any one of the 2013 WHO clinical definitions of severe pneumonia plus hypoxaemia were enrolled between 14/02/17 and 28/02/20 into either the severe hypoxaemia stratum (SpO_2_ < 80%) or the hypoxaemia stratum (SpO_2_ 80–91%). In the severe hypoxaemia stratum, eligible children were randomised (ratio 1:1) to HFNT via OptiFlow or LFO (standard care). In the hypoxaemia stratum, eligible children were randomised (ratio 1:1:2) to HFNT via OptiFlow, LFO (standard care) or no immediate oxygen (permissive hypoxaemia) [9]. Informed written consent was obtained from parents/legal guardians before randomisation. If prior written consent could not be obtained, ethics committees approved verbal consent, and delayed written consent was obtained as soon as practicable. The trial statistician generated and kept a sequential randomised list generated using variably sized permuted blocks, stratified by trial centre. Randomisation packs were consecutively numbered and contained randomised links to opaque sealed envelopes to ensure allocation concealment [13]. All interventions were open, since it was not possible to blind the research staff to any of the interventions as each required its own standard operation protocol for delivery.

The delivery method for HFNT was by AIRVO 2 (Fisher and Paykel Healthcare, Auckland, New Zealand). The AIRVO 2 device is a humidifier with integrated flow generator that delivers to spontaneous breathing patients high flow warmed and humidified air/oxygen blend, thus permitting it to deliver respiratory support on room air alone. HFNT was initiated on FiO_2_ of 21% (room air) with flow rates increase and oxygen titrated in using a structured protocol. Full details of AIRVO 2 are provided in the User Manual: https://resources.fphcare.com/content/airvo-2-user-manual-ui-185045495.pdf

#### Standard care and interventions.

The care provided in the trial consisted of HFNT, LFO and permissive hypoxaemia. The delivery method for LFO depended on local preference but generally was by nasal prongs or by mask. Infants started on a flow rate of 1 L/min and children >1 year commenced on 2 L/min O_2_. The flow rates were titrated up over the first 30 minutes to 1 hour against oxygen saturation (to achieve SpO_2_ ≥ 92%) to a maximum of 2L/min in infants and 4 L/min in children if using nasal prongs. If higher rates were required, children/infants were switched to mask and oxygen gradually increased to 15 L/min depending on type of mask and titrating to response of saturation measurements.

Children in the active intervention arms received oxygen up to 48 hours according to the flow targets listed above. Children remained off HFNT/LFO if SpO_2_ ≥ 92% but weaning would be discontinued or the active intervention restarted again if SpO_2_ fell to <92% before 48 hours. At 48 hours, children still on HFNT requiring supplemental oxygen were switched onto LFO. In the Control (permissive hypoxaemia) arm, children were monitored in accordance to the protocol (extra observations were permitted) and if SpO_2_ (recorded over 5 minutes) fell to < 80%, per protocol oxygen therapy was initiated and the continued in the trial as per the LFO protocol (see Fig A in S1 Text for trial flow diagram).

A total of 1842 eligible children were enrolled into COAST; within the severe hypoxaemia stratum, 194 children were randomised to HFNT and 194 to LFO, and in the hypoxaemia stratum, 363 were randomised to HFNT, 364 to LFO and 727 to permissive hypoxaemia (see Fig A in S1 Text).

### 2.3. Resource use and cost

The cost analysis adopted a hospital perspective and included costs for the 28 days following randomisation. We collated information on those resource use items anticipated to drive major cost differences between the comparator groups. The following resource use items were included: oxygen delivery, medications, blood and fluid products, diagnostic tests, point of care tests, hospital admission and hospital length of stay. The cost of oxygen delivery, medications, blood and fluid, diagnostic tests, point of care tests were calculated for up to 48 hours post-randomisation as per the planned trial interventions. Costs associated with the first and subsequent hospital admissions, additional oxygen beyond 48 hours in the first admission, bed days, fluid and blood transfusion were calculated up to 28 days post-randomisation.

#### Oxygen delivery cost drivers.

The cost of oxygen delivery included costs for oxygen delivery equipment (AIRVO 2, concentrator or oxygen cylinders, electricity usage, use of pulse oximeter, and consumables—face mask and tube only for low flow arm). The costs of oxygen delivery equipment were estimated as cost per patient, assuming two oxygen concentrators, two AIRVO 2 devices, two oxygen cylinders and two pulse oximeters provided to each site. List prices of AIRVO 2 and concentrator were obtained from the manufacturer (in confidence). We calculated per patient costs of AIRVO 2/concentrator assuming the lifetime of equipment was five years and a throughput of 350 eligible patients a year in each site. In COAST, the expected delivery mode of oxygen for HFNT was AIRVO 2 combined with an oxygen concentrator and for LFO it was an oxygen concentrator. However, the trial case report forms (CRFs) did not specify the oxygen delivery mode, and for either randomised arm, oxygen may have been delivered using either a cylinder or concentrator. In calculating the cost of oxygen delivery for either HFNT or LFO, we assumed that, in each arm, for 50% of participants oxygen was delivered by an oxygen cylinder and for 50% by a concentrator. We varied these proportions in sensitivity analyses (see later section).

The oxygen concentrator either used by itself or plugged into an AIRVO 2 device did not require any oxygen from the oxygen cylinders. Oxygen consumption from a cylinder in the HFNT (Airvo+cyl) or LFO arm (cyl) was calculated from individual-level data on oxygen volume and duration from the trial CRFs as:


$$O_{\text{2}}Litres_{Airvo+cyl}=\frac{HrsOnTrt*\left((FlowRatePerHr\;*FiO_{\text{2}}\%\right)-(FlowRatePerHr*\text{21}))}{\left(\text{100}-\text{21}\right)}$$

$$O_{\text{2}}Litres_{cyl}\;=HrsOnTrt\;*\left(\;FlowRatePerHr\;*FiO_{\text{2}}\%/\text{100}\right)$$

Electricity costs per day were calculated using the volume and duration oxygen use data. AIRVO 2 electricity use was allowed to depend on the flow rate with KWh = (2.12667 + 0.06333*flow rate)/48 for flow rate measured in L/min. The concentrator used in the trial, AirSep Newlife intensity, used 0.41KWh of electricity to run, regardless of flow rate used. Oxygen delivered solely using a cylinder did not require electricity. Cylinder refills at each site were estimated based on the total amount of oxygen consumed and deducting the volume of oxygen from the two full cylinders provided at the start of the trial.

#### Blood, fluid and medications.

The trial CRFs collected information on the use of fluids and blood products (dextrose products, whole blood, red blood cells (RBC), normal saline products and ringers lactate products) given to each trial participant. It was assumed that blood packs are not reusable if not fully administered to a patient.

The cost analysis included the following medications as these were frequently used: Gentamicin, Ampicillin, Ceftriaxone, Hydrocortisone, Salbutamol and Prednisolone. For each child the number of prescribed medications and volume of medications during hospital index admissions was extracted from the trial CRFs. Costs were provided by each of the study sites.

#### Diagnostic tests, point of care test and other costs.

We included the costs of those diagnostic and other tests that were frequently used and possibly related to the interventions, these were: diagnostic tests (Hb, Malaria RDT, sodium, potassium, urea, creatinine and pathogen count estimated using a blood culture (BACTEC)); point of care tests (lactate, glucose, and HIV); other costs (x-ray and cost of admitting a patient (consumables and file)). Costs were provided by each of the study sites.

#### Hospital length of stay (LOS).

The LOS during the index admission and any readmissions was extracted from the trial CRFs, and used to calculate the ‘bed-day’ costs of the hospital stay including meals.

#### Unit costs and total costs.

The resource use data, provided by the study sites for each expenditure during the time of the study, were valued using appropriate unit costs (Table 1) to estimate total costs per patient for each intervention group. Costs were collected after the trial ended, and were initially collated in local currency in Kenya (KES) and Uganda (UGX) in 2021 prices. They were converted to US dollars using 2021 purchasing parity conversion factors with $1 = 109.64 KES [17] or 3,587.05 UGX [18], and inflated to 2024 prices using GDP deflator [19].

**Table 1:** Unit cost ($), costs are reported in 2024 prices.

Product	Unit/ Description	Cost (USD)	
***Equipment/cylinders/electricity***			
***High flow Arm***			
AIRVO 2	Cost per patient	184.53	
AIRVO 2 electricity costs (per day)	AIRVO 2 only	0.18	
	AIRVO 2 + concentrator	0.36	
***Low flow Arm***			
Oxygen concentrator	Cost per patient	2.76	
Concentrator electricity	Per day	0.18	
Low flow: Face mask	Single use, per piece	2.20	
Low flow: Tube	Single use, per piece	9.91	
***Both Arms***			
Oxygen cylinder	Cost per patient	2.76	
Oxygen cylinder refill	6,600L	60.33	
Pulse oximeter	Cost per patient	6.77	
***Medication***			
Gentamicin	Ampoule (80mg)	0.06	
Ampicillin	Vial (500mg)	0.19	
Ceftriaxone	Vial (1000mg)	2.43	
Hydrocortisone	Vial (100mg)	0.25	
Salbutamol/Ventolin	Vial (10ml)	5.90	
Prednisolone	1 tab	0.01	
***Blood & Fluids***			
Dextrose products	Bottle (500ml)	0.42	
Blood products	Whole blood (450ml)	120.66	
	RBC (150ml)	52.67^*^	
Normal saline products	Bottle (500ml)	0.45	
Ringers lactate products	Bottle (500ml)	0.42	
***Diagnostic Tests***			
FBC (Hb)	Cost per test	6.85	
Malaria RDT		1.97	
Sodium		4.95	
Potassium		4.95	
Urea bun		2.06	
Creatinine		2.93	
Blood culture (BACTEC)		16.59	
***Point of care tests***			
Lactate	Cost per test	3.25	
Glucose		0.17	
HIV		0.87	
***Other Costs***			
X-ray (pneumonia)	Per image	4.95	
		**Kenya**	**Uganda**
Hospital Admission	Consumables	5.50	0.00
	File	1.65	0.00
***Bed day costs***			
Hospital bed day	Bed	17.86	10.71
	Meals	0.82	0.00

* RBC (450ml; $158) were assumed to be split into 3 pedipacks and the cost was adjusted accordingly.

Actual costs came from communication with the trial sites.

### 2.4. Analysis

In this study, we conducted a cost consequence analysis. This is a suitable approach, recommended by NICE, for the evaluation of complex interventions when different outcomes cannot be combined into a single index measure [16]. For each stratum, we reported the costs per patient for each of the randomised arms and we also presented the incremental costs, defined as the difference in mean total costs, between the respective strategies of interest. For the severe hypoxaemia stratum, the incremental costs were then estimated as the differences in these mean costs for those randomised to HFNT compared with LFO (standard care). In the hypoxaemia stratum, when calculating the incremental costs, we calculated mean costs pooled across individuals in the HFNT and LFO arms and defined these pooled mean costs of both oxygenation strategies as the ‘liberal oxygenation strategies’ and compared these to the mean for the permissive hypoxaemia arm.

We applied a regression model to report incremental costs for HFNT versus LFO (severe hypoxaemia) and for liberal oxygenation vs permissive hypoxaemia (hypoxemia) adjusting for between-arm differences in baseline SpO_2_ and trial centre as per the primary clinical analysis of COAST [20]. As the distribution of costs was right-skewed, a small proportion of patients had relatively high costs, we choose to apply a generalised linear regression model that assumed the residuals were drawn from a Gamma distribution. Confidence intervals around incremental costs were estimated using the non-parametric bootstrap with 500 replications [21].

#### Health consequences.

As per cost-consequence guidelines [16], we presented estimates of the effects of the primary and main secondary clinical endpoints at 28 days post-randomisation, alongside the costs. The clinical endpoints were all-cause mortality (primary), weight for age z-score and mid-upper arm circumference z-score (secondary) [13]. For each stratum, we reported the absolute levels for each randomised arm according to adjusted means. We followed the cost analysis in the reporting of the incremental effects of the alternative strategies on these clinical endpoints.

#### Sensitivity analyses.

As in any health economic evaluation, we were required to make assumptions. We therefore undertook sensitivity analyses to investigate the impact on the results of making different assumptions to the base case. In the sensitivity analyses, we (a) deterministically varied the proportion of participants for whom oxygen was supplied by concentrator rather than cylinders between 0.25 and 0.75 (compared to 0.50, base case) (b) assumed a Normal rather than a Gamma distribution for the residuals in the regression models estimating incremental costs and (c) restricted the time horizon for the inclusion of costs to the first 48 hours from randomisation (rather than 28 days).

## 3. Results

### 3.1. Patient characteristics

Table 2 presents the baseline characteristics for each randomised arm within each stratum. The baseline characteristics were all balanced across the randomised arms.

**Table 2:** Baseline characteristics of study population [13].

Characteristics	Severe hypoxaemia stratum (SpO_2_ < 80%)			Hypoxaemia stratum (SpO_2_ 80 to <92%)		
	HFNT (N = 194)	LFO (N = 194)		HFNT (N = 363)	LFO (N = 364)	Permissive (N = 727)
Age (months): median (IQR)	7 (2, 21)	7 (2, 16)		9 (4, 24)	9 (4, 22)	10 (4, 25)
Sex (Male): n (%)	93 (47.9)	97 (50.0)		213 (58.7)	214 (58.8)	422 (58.0)
SpO_2_: median (IQR)	75 (68, 78)	75 (66, 77)		88 (86, 89)	88 (86, 89)	88 (86, 90)
SpO_2_ < 70% or <85%: n(%)	55 (28.4)	60 (30.9)		60 (16.5)	65 (17.9)	98 (13.5)
Weight (Kg): median (IQR)	6.8 (4.8, 10)	6.6 (4.8, 9.0)		8.1 (6.4, 11.0)	7.9 (6.2, 10.4)	8.3 (6.5, 10.8)
MUAC (cm): median (IQR)	13.0 (11.4, 14.2)	13.0 (11.5, 14.2)		14.0 (13.0, 15.0)	13.7 (12.7, 14.7)	14.0 (12.8, 15.0)
Respiratory rate: median (IQR)	65 (56–79)	66.5 (56–79)		61 (52–69)	60 (52–68)	60 (51–67)
White cell count (× 10^3^/μL): median (IQR)	13.9 (9.5–20.3)	13.2 (9.4–18.7)		12.5 (9.2–17.3)	11.9 (8.3–16.4)	11.9 (8.3–17.9)
N (%)						
Fever (> 35 °C)	105 (54.1)	94 (48.5)		191 (52.6)	188 (51.6)	341 (46.9)
Hypothermia (< 36 °C)	5 (2.6)	13 (6.7)		4 (1.1)	9 (2.5)	20 (2.8)
Tachypnoea	178 (91.8)	176 (90.7)		330 (90.9)	331 (90.9)	654 (90.0)
Indrawing	186 (95.9)	187 (96.4)		334 (92)	343 (94.2)	658 (90.5)
Cyanosis	13 (6.8)	15 (7.7)		3 (0.8)	3 (0.8)	4 (0.6)
Crepitations	136 (70.1)	149 (76.8)		271 (74.7)	267 (73.4)	530 (72.9)
Wheeze	42 (21.6)	37 (19.1)		91 (25.1)	93 (25.5)	182 (25.0)
Pneumonia	128 (66.0)	113 (58.2)		227 (62.5)	217 (59.6)	426 (58.6)
Severe tachycardia	72 (37.1)	77 (39.7)		90 (24.8)	100 (27.5)	179 (24.6)
Compensated shock	118	121 (62.4)		139 (38.3)	145 (39.8)	287 (39.5)
Severe pallor	31 (16.0)	24 (12.4)		36 (9.9)	26 (7.1)	57 (7.8)
Vomiting/diarrhoea	62 (32.0)	67 (34.5)		120 (33.1)	136 (37.4)	239 (32.9)
Dehydrated	11 (5.7)	20 (10.3)		11 (3)	15 (4.1)	22 (3.0)
Conscious level: responds to						
Pain or voice	33 (17)	24 (12.4)		14 (3.9)	14 (3.8)	26 (3.6)
Unresponsive	8 (4.1)	13 (6.7)		2 (0.6)	3 (0.8)	3 (0.4)
Severely malnourished	19 (9.8)	29 (14.9)		10 (2.8)	24 (6.6)	33 (4.5)
Sickle cell disease	10 (5.2)	7 (3.6)		26 (7.2)	26 (7.1)	40 (5.5)
Developmental delay	16 (8.2)	16 (8.2)		21 (5.8)	17 (4.7)	40 (5.5)
Haemoglobin (g/dL): median (IQR)	9.6 (7.3, 11.1)	10.2 (8.7, 11.3)		10.2 (8.8, 11.4)	10.3 (8.9, 11.4)	10.4 (8.9, 11.7)
Severe anaemia (Hb < 5/dl)	24 (12.4)	13 (6.7)		33 (9.1)	26 (7.1)	59 (8.1)
Leucocytosis	120 (61.9)	117 (60.3)		204 (56.2)	193 (53.0)	388 (53.4)
HIV	6 (3.1)	11 (5.7)		4 (1.1)	15 (4.1)	13 (1.8)
Malaria RDT	25 (12.9)	18 (9.3)		49 (13.5)	38 (10.4)	98 (13.5)
Malaria slide positive^a^	11 (5.7)	13 (6.7)		26 (7.2)	15 (4.1)	36 (5.0)
Bacteraemia	10 (5.2)	7 (3.6)		8 (2.2)	8 (2.2)	19
Hypoglycaemia (glucose < 3/mmol/L)	10 (5.2)	9 (4.6)		7 (1.9)	5 (1.4)	21 (2.9)
Lactate > 5 mmol/L	41 (21.1)	38 (19.6)		34 (9.4)	21 (5.8)	54 (7.4)
Antibiotics in illness	112 (57.7)	121 (62.4)		205 (56.5)	201 (55.2)	404 (55.6)
Antimalarial in Illness	41 (21.1)	42 (21.6)		97 (26.7)	85 (23.4)	169 (23.2)
Missing values for each arm: Severe High flow; Severe Low flow; Hypoxaemia High flow; Hypoxaemia Low flow; Hypoxaemia Permissive						
Tachypnoea: 0 (0.0); 0 (0.0); 0 (0.0); 0 (0.0); 1 (0.1) Indrawing: 1 (0.5); 1 (0.5); 0 (0.0); 0 (0.0); 0 (0.0) Cyanosis: 2 (1.0); 0 (0.0); 0 (0.0); 0 (0.0); 0 (0.0) Crepitations: 2 (1.0); 0 (0.0); 0 (0.0); 0 (0.0); 4 (0.5) Wheeze: 3 (1.5); 0 (0.0); 1 (0.2); 0 (0.0); 4 (0.5) Pneumonia: 29 (14.9); 39 (20.1); 16 (4.4); 22 (6.0); 32 (4.4) Severe tachycardia: 0 (0.0); 0 (0.0); 1 (0.2); 0 (0.0); 0 (0.0) Compensated shock: 1 (0.5); 0 (0.0); 0 (0.0); 0 (0.0); 0 (0.0) Severe pallor: 1 (0.5); 0 (0.0); 0 (0.0); 0 (0.0); 0 (0.0) Vomiting/diarrhoea: 1 (0.5); 0 (0.0); 1 (0.2); 0 (0.0); 1 (0.1) Dehydrated: 3 (1.5); 0 (0.0); 0 (0.0); 1 (0.3); 2 (0.3) Conscious level: 0 (0.0); 0 (0.0); 0 (0.0); 1 (0.3); 0 (0.0)			Severely malnourished: 1 (0.5); 0 (0.0); 0 (0.0); 2 (0.5); 1 (0.1) Developmental delay: 1 (0.5); 0 (0.0); 0 (0.0); 2 (0.5); 0 (0.0) Severe anaemia (Hb < 5/dl): 10 (5.2); 12 (6.2); 11 (3.0); 16 (4.4); 29 (4.0) Leucocytosis: 10 (5.2); 12 (6.2); 12 (3.3); 17 (4.7); 29 (4.0) HIV: 6 (3.1); 6 (3.1); 9 (2.5); 8 (2.2); 20 (2.8) Malaria RDT: 13 (6.7); 13 (6.7); 13 (3.6); 12 (3.3); 27 (3.7) Malaria slide positive: 7 (3.6); 12 (6.2); 11 (3.0); 10 (2.7); 27 (3.7) Bacteraemia: 7 (3.6); 11 (5.7); 9 (2.5); 11 (3.0); 22 (3.0) Hypoglycaemia (glucose < 3/mmol/L): 2 (1.0); 1 (0.5); 0 (0.0); 0 (0.0); 0 (0.0) Lactate > 5 mmol/L: 3 (1.5); 4 (2.1); 9 (2.5); 6 (0.8); 12 (1.7) Antibiotics in illness: 8 (4.1); 2 (1.0); 5 (1.4); 6 (0.8); 6 (0.8) Antimalarial in illness: 6(3.1); 4 (2.1); 2(0.6), 1(0.3); 1 (0.1)			

### 3.2. Resource use

#### Oxygen delivery.

In the severe hypoxaemia stratum, the mean duration of oxygen support was similar across the arms with 29.85 hours (HFNT) and 27.97 hours (LFO), but with a lower average volume of oxygen support in the HFNT (3,183 L) compared to the LFO (3,364 L) (Table 3). In the hypoxaemia stratum, the mean duration and volume of oxygen support were similar for both liberal oxygenation strategies (HFNT, 16.94 h and 1,227 L; LFO, 15.89 h and 1,360 L), but the corresponding means were lower (3.51 h and 338 L) for the permissive hypoxaemia arm.

**Table 3:** Resource use up to 28 days, Mean (SD).

	Severe hypoxaemia stratum (SpO_2_ < 80%)		Hypoxaemia stratum (SpO_2_ 80 to <92%)		
	HFNT (N = 194)	LFO (N = 194)	HFNT (N = 363)	LFO (N = 364)	Permissive (N = 727)
**O**_**2**_ **support within 48 hours**					
Hours	30.56 (18.38)	28.34 (18.65)	19.91 (16.65)	18.29 (16.26)	6.87 (11.20)
Volume (Litre)*	3,201 (3,401)	3,355 (3,986)	1,225 (2,392)	1,360 (2,268)	338 (1,210)
**Blood and fluid**					
RBC (bag = 150ml)	0.12 (0.64)	0.09 (0.42)	0.16 (0.74)	0.11 (0.61)	0.11 (0.68)
Whole blood (bag = 450ml)	0.10 (0.34)	0.06 (0.24)	0.12 (0.42)	0.13 (0.58)	0.12 (0.47)
Follow-up blood (units)	0.01 (0.07)	0.01 (0.07)	0.27 (3.82)	0.02 (0.27)	0.00 (0.00)
Dextrose/saline/ringers	0.14 (0.35)	0.23 (0.42)	0.09 (0.29)	0.10 (0.30)	0.12 (0.32)
**Medications**					
Gentamicin	0.74 (0.44)	0.75 (0.43)	0.82 (0.39)	0.84 (0.36)	0.84 (0.37)
Ampicillin	0.41 (0.49)	0.41 (0.49)	0.45 (0.50)	0.46 (0.50)	0.47 (0.50)
Ceftriaxone	0.47 (0.50)	0.46 (0.50)	0.37 (0.48)	0.38 (0.49)	0.37 (0.48)
Hydrocortisone	0.29 (0.46)	0.28 (0.45)	0.38 (0.49)	0.36 (0.48)	0.38 (0.49)
Salbutamol/Ventolin	0.14 (0.35)	0.15 (0.36)	0.13 (0.34)	0.15 (0.36)	0.15 (0.35)
Prednisolone	0.03 (0.17)	0.04 (0.19)	0.02 (0.16)	0.03 (0.17)	0.04 (0.19)
Artesunate	0.16 (0.37)	0.13 (0.34)	0.18 (0.38)	0.17 (0.37)	0.18 (0.39)
Artemether lumetantrine	0.00 (0.00)	0.01 (0.07)	0.00 (0.05)	0.00 (0.05)	0.01 (0.08)
Coartem	0.05 (0.22)	0.04 (0.19)	0.07 (0.26)	0.05 (0.23)	0.07 (0.26)
**Diagnostic tests**					
FBC (Hb)	0.95 (0.22)	0.94 (0.24)	0.97 (0.17)	0.96 (0.21)	0.96 (0.19)
Malaria RDT	0.97 (0.17)	0.93 (0.25)	0.96 (0.19)	0.97 (0.18)	0.97 (0.18)
Sodium	0.18 (0.39)	0.18 (0.38)	0.11 (0.31)	0.11 (0.31)	0.10 (0.30)
Potassium	0.18 (0.39)	0.18 (0.38)	0.11 (0.31)	0.11 (0.31)	0.10 (0.30)
Urea/BUN	0.18 (0.38)	0.15 (0.36)	0.10 (0.31)	0.10 (0.30)	0.09 (0.29)
Creatinine	0.18 (0.39)	0.18 (0.38)	0.11 (0.31)	0.11 (0.31)	0.10 (0.30)
Blood culture (BACTEC)	0.96 (0.19)	0.94 (0.23)	0.98 (0.16)	0.97 (0.17)	0.97 (0.17)
**Point of care tests**					
Lactate	0.98 (0.12)	0.99 (0.10)	0.99 (0.12)	0.99 (0.12)	0.98 (0.13)
Glucose	0.99 (0.07)	1.00 (0.00)	1.00 (0.00)	1.00 (0.00)	1.00 (0.00)
HIV	0.98 (0.14)	0.98 (0.14)	0.98 (0.13)	0.98 (0.14)	0.97 (0.16)
**Other costs**					
X-ray (pneumonia)	0.15 (0.36)	0.20 (0.40)	0.04 (0.21)	0.06 (0.23)	0.04 (0.21)
Number of hospital admissions	1.09 (0.46)	1.04 (0.27)	1.08 (0.37)	1.07 (0.35)	1.08 (0.37)
**Bed days**	6.92 (5.15)	6.71 (5.36)	5.50 (3.79)	5.62 (3.65)	5.26 (3.18)

* Assuming 100% cylinder use to supply O_2_ in the high and low flow arms.

#### Blood, fluid and medications.

In the severe hypoxaemia stratum, the mean number of RBC and whole blood bags received were higher for the HFNT (0.12 and 0.10 bags) compared to the LFO arm (0.09 and 0.06 bags). For both the severe hypoxaemia and hypoxaemia strata, the mean use of medications was similar across the arms.

#### Diagnostic tests, point of care test and other costs.

For both strata, the mean number of diagnostics tests for HB, malaria RDT, and pathogen (using blood cultures) were relatively high (about 1 per patient) and similar across arms. The number of other diagnostics tests (sodium, potassium, urea, and creatinine) per patient were relatively low (0.20 per patient) and similar across arms in both strata. The number of point-of-care tests (lactate, glucose, and HIV) and x-rays were similar across arms in both strata.

#### Hospital LOS (Bed days).

In the severe hypoxaemia stratum, the mean LOS was 6.90 days (HFNT) and 6.71 days (LFO), respectively, and in the hypoxaemia stratum, 5.50 days (HFNT), 5.62 days (LFO) and 5.26 days (permissive).

### 3.3. Costs

In both strata, the fixed and consumable costs of equipment account for the majority of the total intervention costs for HFNT compared with the other strategies (Table 4). In the severe hypoxaemia stratum, the mean equipment costs for the HFNT arm were $189.93 versus $21.64 for the LFO arm, and in the hypoxaemia stratum the corresponding mean costs were $190.44 (HFNT), $22.11 (LFO) and $9.00 (permissive), respectively. For both strata, the costs of oxygen consumption were similar for the HFNT and LFO arms, and low for the permissive arm in the hypoxaemia stratum. The cost of electricity was relatively low compared to the other costs for delivering the interventions.

**Table 4:** Equipment, O_2_, electricity costs ($) up to 48 hours, Mean (SD).

	Severe hypoxaemia stratum (SpO_2_ < 80%)		Hypoxaemia stratum (SpO_2_ 80 to <92%)		
	HFNT (N = 194)	LFO (N = 194)	HFNT (N = 363)	LFO (N = 364)	Permissive (N = 727)
Equipment (fixed)	13.24 (0.84)	9.53 (0)	13.20 (0.56)	9.54 (0.19)	7.18 (0.99)
Equipment (consumables)	176.69 (18.22)	12.11 (0)	177.24 (13.27)	12.57 (8.70)	1.82 (4.33)
O_2_ consumption	19.25 (25.80)	18.82 (20.83)	8.30 (10.85)	9.06 (11.34)	4.30 (5.52)
Electricity consumption	1.32 (0.94)	1.09 (1.02)	0.55 (0.71)	0.59 (0.62)	0.13 (0.39)
**Total Equipment, O**_**2**_ **, electricity costs**	**210.52** **(34.42)**	**41.55** **(21.53)**	**199.31** **(18.45)**	**31.74** **(14.56)**	**13.43** **(10.23)**

In both strata, the intervention costs were the main cost driver for the HFNT (Table 5). In both strata, the mean costs for blood and fluid were higher for the HFNT arm compared with the other strategies, although these mean estimates are highly uncertain. All other mean costs were similar across the arms.

**Table 5:** Costs ($) up to 28 days unless otherwise stated, Mean (SD), 2021 price year.

Resource type	Severe hypoxaemia stratum (SpO_2_ < 80%)		Hypoxaemia stratum (SpO_2_ 80 to <92%)		
	HFNT (N = 194)	LFO (N = 194)	HFNT (N = 363)	LFO (N = 364)	Permissive (N = 727)
Equipment, O_2_, electricity (48 hours)	210.52 (34.42)	41.55 (21.53)	199.31 (18.45)	31.74 (14.56)	13.43 (10.23)
Blood & Fluids	19.38 (52.29)	13.09 (41.48)	54.43 (452.38)	23.81 (99.12)	20.31 (71.67)
Medications	6.12 (8.95)	12.45 (101.50)	5.53 (9.10)	9.60 (65.34)	5.19 (7.00)
Diagnostic Tests	38.02 (9.40)	36.74 (9.71)	37.73 (7.41)	37.29 (7.66)	37.37 (8.00)
Point of care tests	17.97 (5.26)	18.14 (6.05)	18.11 (3.25)	18.18 (3.53)	18.14 (3.38)
Other costs	2.95 (5.20)	2.76 (3.77)	1.72 (3.81)	1.66 (3.38)	1.70 (3.74)
Bed day	98.07 (82.35)	94.00 (78.27)	75.12 (56.39)	75.98 (50.50)	71.65 (48.12)
**Total Costs**	**393.04** **(119.76)**	**218.73** **(141.73)**	**391.95** **(488.35)**	**198.26** **(149.63)**	**167.80** **(98.50)**

**Fig 1:**
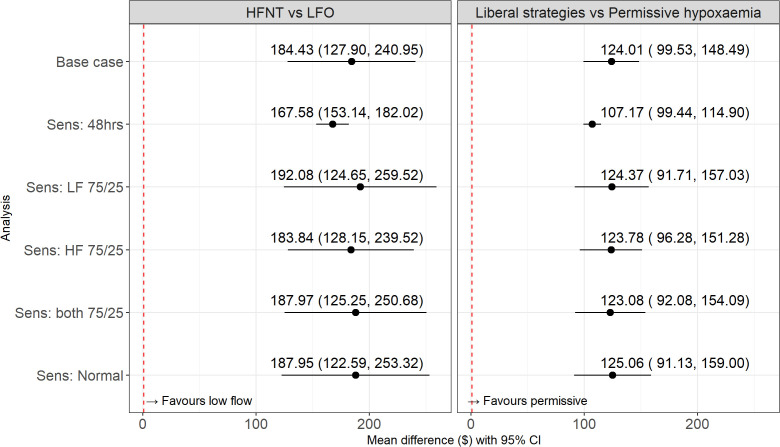
Incremental effects (with 95% CI) for Total cost for the base case and all sensitivity analyses (N = 1,841). LFO: Low flow oxygen; HFNT: High flow nasal therapy; Sens: Sensitivity Analysis. Sens:48hrs- Total costs restricted to those occurring within the first 48 hours; Sens: LF 75/25 – the proportion of oxygen supplied by concentrator versus cylinder in the low flow arm was 75% vs. 25%, compared to 50/50 in the high flow arm; Sens: HF 75/25 – the proportion of oxygen supplied by concentrator versus cylinder in the high flow arm was 75% vs. 25%, compared to 50/50 in the low flow arm; Sens: both 75/25 – the proportion of oxygen supplied by concentrator versus cylinder in both high and low flow arms is 75% vs. 25%; Sens: Normal- Changed the distributional assumption from Gamma to Normal.

For the severe hypoxaemia stratum, the adjusted incremental cost per patient of HFNT versus LFO was $184.43 (95% CI $127.90 to $240.95) (Fig 1, Table A in S1 Text). In the hypoxaemia stratum, the adjusted incremental cost of liberal strategies (HFNT and LFO combined) versus the permissive hypoxaemia strategy was $124.01 (95% CI $99.53 to $148.49). For HFNT versus permissive, the adjusted cost difference was $216.22 (95% CI 160.77 to 271.68), and for LFO versus permissive it was $31.80 (95% CI 11.49 to 52.11). The sensitivity analyses found that in scenarios that made alternative assumptions to the base case, the estimated incremental costs were similar, and so the base case results were robust to the assumptions made in the main analysis. Data available in S1 Data.

### 3.4. Health outcomes

Within the severe hypoxaemia stratum, 36 (18.6%) patients in the HFNT arm had died by 28 days, compared to 45 (23.4%) in the LFO arm, with an adjusted odds ratio of 0.75 (95% CI 0.49 to 1.16; Table 6). In the hypoxaemia stratum by 28 days, 12 (3.3%), 15 (4.1%) and 28 (3.9%) died from the HFNT, LFO and permissive arms, respectively. The adjusted mean difference between weight for age z-score was 0.05 (95% CI -0.05 to 0.16) for HFNT versus LFO (severe hypoxaemia stratum) and 0.04 (95% CI -0.05 to 0.13) for liberal strategies versus permissive hypoxaemia (hypoxaemia stratum). The adjusted mean difference between mid-upper arm circumference z score was 0.08 (95% CI -0.11 to 0.27) for HFNT versus LFO and 0.02 (95% CI -0.14 to 0.17) for liberal strategies versus permissive hypoxaemia. Within the severe hypoxaemia stratum, the mean length of stay was 7.0 days (SD 5.7) for HFNT and 6.8 days (SD 6.1) for LFO. Within the hypoxaemia stratum, the mean length of stay was 5.9 days (SD 10.3) for HFNT, 5.6 days (SD 3.7) for LFO, and 5.1 days (SD 3.0) for permissive hypoxaemia. The adjusted mean difference was 0.22 days (95% CI -0.91 to 1.37) for HFNT versus LFO and 0.61 days (95% CI 0.42 to 0.80) for liberal strategies versus permissive hypoxaemia. Within the severe hypoxaemia stratum, 10 patients had a readmission (5.2%) in the HFNT arm and 5 (2.6%) in LFO arm. Within the hypoxaemia stratum, 20 (5.5%) patients on HFNT, 18 (5.0%) on LFO and 36 (5.0%) on permissive hypoxaemia had a hospital readmission. The adjusted odds ratio of readmission for HFNT versus LFO was 1.33 (95% CI 0.62 to 2.85), and 1.05 (0.53, 2.07) for liberal strategies versus permissive hypoxaemia.

**Table 6:** Cost and consequences at 28 days: total costs ($), mortality, weight for age z-score, and mid-upper arm circumference z-score [13]. All numbers are Mean (SD), unless stated otherwise.

	Severe hypoxaemia stratum (SpO_2_ < 80%)		Hypoxaemia stratum (SpO_2_ 80 to <92%)			Incremental effects (adjusted) Mean/OR* (95% CI)	
	HFNT (N = 194)	LFO (N = 194)	HFNT (N = 363)	LFO (N = 364)	Permissive (N = 727)	HFNT vs LFO	Liberal vs Permissive
Total Cost ($)	393.04 (119.76)	218.73 (141.73)	391.95 (488.35)	198.26 (149.63)	167.80 (98.50)	184.43 (127.90, 240.95)	124.01 (99.53, 148.49)
						**HFNT vs permissive**	**LFO vs Permissive**
						216.22 (160.77, 271.68)	31.80 (11.49, 52.11)
Mortality* N (%)	36 (18.6)	45 (23.4)	12 (3.3)	15 (4.1)	28 (3.9)	0.75 (0.49, 1.16)	0.92 (0.53, 1.59)
Weight for age z score	-0.4 (1.8)	-0.6 (1.8)	0 (1.7)	-0.2 (1.6)	-0.2 (1.6)	0.05 (-0.05, 0.16)	0.04 (-0.05, 0.13)
Mid-upper arm circumference z score	-0.8 (1.3)	-0.7 (1.4)	-0.1 (1.4)	-0.3 (1.3)	-0.2 (1.3)	0.08 (-0.11, 0.27)	0.02 (-0.14, 0.17)
Length of stay within first 28 days	7.0 (5.7)	6.8 (6.1)	5.9 (10.3)	5.6 (3.7)	5.1 (3.0)	0.22 (-0.91, 1.37)	0.61 (0.42, 0.80)
Readmission N (%)	10 (5.2)	5 (2.6)	20 (5.5)	18 (5.0)	36 (5.0)	1.33 (0.62, 2.85)	1.05 (0.53, 2.07)

*Adjusted odds ratio for mortality;

OR: Odds ratio; CI: confidence interval; SD: standard deviation.

## 4. Discussion

The economic analysis alongside COAST, which demonstrated no evidence of a difference in health outcomes [13] (including mortality), found that treatment with a liberal oxygen strategy (either HFNT or LFO) for children with hypoxaemia is more costly than permissive hypoxaemia (mean cost difference $124.01, 95%CI $99.53 to $148.49). For children with severe hypoxaemia, HFNT is more expensive than LFO (mean cost difference $184.43, 95% CI $127.90 to $240.95) with no significant differences in clinical outcomes between the intervention groups, and similar duration of therapy and oxygen volume between the groups. The main driver of cost differences for liberal oxygen therapy is the high cost of initial equipment and consumables, particularly the equipment in the HFNT arm, while other costs are similar across treatment groups in both strata. The sensitivity analysis finds that this conclusion is robust to alternative assumptions to those made in the base-case analysis.

When there are significant gaps between supply and demand of oxygen [10], and where the upkeep of equipment operational quality is costly [22], the use of the less costly interventions which may result in similar health outcomes is important. Cost-effectiveness evidence in this context has been scarce to date. A systematic literature review in LMIC [23] found that in general interventions to strengthen oxygen systems are likely to reduce hospital based pneumonia mortality in children and these interventions are likely to be cost-effective. The four studies included in the review have calculated costs from a facility level perspective using observational data. The studies found, similarly to our own study, that the major drivers of costs were equipment costs accounting between 65% and 73% of total costs. The review also reported that strengthening oxygen systems reduce hospital-based pneumonia deaths by nearly half and hospital-based paediatric deaths overall by a quarter, but did not provide any evidence to inform which strategies would be able to maintain outcomes while reducing costs, nor provide any insights into how to target strategies according to particular stratum of patients. However, COAST didn’t find any significant impact of liberal oxygen therapy on a range of health outcomes, including mortality, weight for age z-score and mid-upper arm circumference z-score, as the trial was stopped prematurely. Nevertheless, the finding of an absolute reduction (of 40%) in 48-h mortality in children receiving HFNT compared to LFO (relative reduction 0.60 (0.33–1.06; p = 0.08) warrants further investigation in a larger well-powered study.

The systematic review also reported median cost-effectiveness of US$68 per disability-adjusted life year (DALY) averted (range: US$44–US$225). The cost-effectiveness results from the literature review are not directly comparable to our study. Our study didn’t measure health-related quality of life, and hence didn’t report quality-adjusted life years or DALYS in our study. This analysis has focused on costing resource use from the COAST trial but implementing these treatments in practice could incur additional costs. The supply of oxygen for resource-limited hospitals is a challenge [10], and other resources, in addition to the purchasing of oxygen supply machines, could be required to ensure facilities are prepared to implement oxygen supplementation approaches. The volumes of oxygen received in the active intervention arms in the trial were much lower than has previously been reported [8], albeit that study was in a general admission cohort of paediatric and neonatal patients. We have recently reported volumes of oxygen received over the initial 48-hour period. Median oxygen use in the hypoxaemia group (79% of participant of study participants) was 480L (IQR 236.2, 2132.2) in LFO am compared to 113.4L (IQR 0.0, 1453.9) in the HFNT [24]. This, we suggest, could be accounted for by the early weaning at 8-hours (55.6%) in the LFO arm versus (45%) when saturations had normalised.

Notably, the HFNT strategy employed in COAST used a mixture of room air and oxygen concentrators/cylinders, reducing the cost burden on health services, with no evidence of harm on patients. Considerations for implementation in practice are outside the scope of this analysis.

This study has several strengths. This study included a prospectively designed economic evaluation integrated with the well-designed COAST trial, which ensured that detailed resource use data were collected for each randomised patient. The economic analysis used detailed patient level resource use data and costed up each resource use item using appropriate methods. This study provides a comprehensive understanding of the cost implications by capturing detailed patient level resource use data for oxygen delivery, blood and fluids, medications, diagnostic tests, point of care test, and hospital inpatient costs. The costs of oxygen delivery equipment per patient were estimated considering a plausible resource use assumption reflecting clinical practice in this context. We have varied these assumptions in sensitivity analyses and the base-case results were not sensitive to these alternative assumptions. The costs and outcome results of our study are based on patient level data collected from multiple sites in Africa and likely to be more generalisable than other published evidence. The study was subject to a few limitations. Our analysis only considered a 28-day time horizon but the relative costs and effects of liberal oxygen therapy may continue over a longer period of time beyond hospital discharge. Our analysis has adopted a health services perspective, and excluded any costs incurred by households which may be considerable in childhood illness. The per patient cost of AIRVO 2 and concentrators assumed 350 eligible patients being admitted to hospital. It is possible that patient admissions for pneumonia could be lower than this which would increase the per patient cost of AIRVO 2 and the oxygen concentrators. In addition, there are other important outcomes beyond clinical health outcomes such as health-related quality of life, which is not captured in our study. Assessing the quality of life for the children and their caregivers is crucial for evaluating relative cost-effectiveness of interventions in children. Finally, as indicated above, a 40% reduction in odds of 48-hour mortality for patients on HFNT versus LFO may have resulted in patients on HFNT surviving longer and using more resources which led to increased costs for this intervention arm. However, due to the trial stopping early this cannot be fully explored.

In conclusion, even though the trial was stopped early, an economic evaluation yielded valuable insights into the cost implications and potential value-for-money of the intervention. This can inform future funding decisions, policy planning, or prioritisation of further research, especially in cases where clinical effects were promising but underpowered.

## Supporting information


S1 Text.
**Fig A:** Flow of patients in COAST. **Table A**: Output from the Gamma regression model.
(DOCX)

S1 Checklist CHEERS checklist. (DOCX)

S2 Checklist Inclusivity-in-global-research-questionnaire. (DOCX)

S1 Data COAST data analysis. (XLSX)
