# Supplementary material for: A cost-consequence analysis of the children’s administration oxygenation strategies trial (COAST) in severe pneumonia
Source: PLOS Glob Public Health. 2026 Jan 8;6(1):e0005654. doi: 10.1371/journal.pgph.0005654 (PMC12782444; doi:10.1371/journal.pgph.0005654)
Supplement: S1 Text. — Fig A: Flow of patients in COAST. Table A: Output from the Gamma regression model. (DOCX) [file pgph.0005654.s001.docx]

# S1 Text


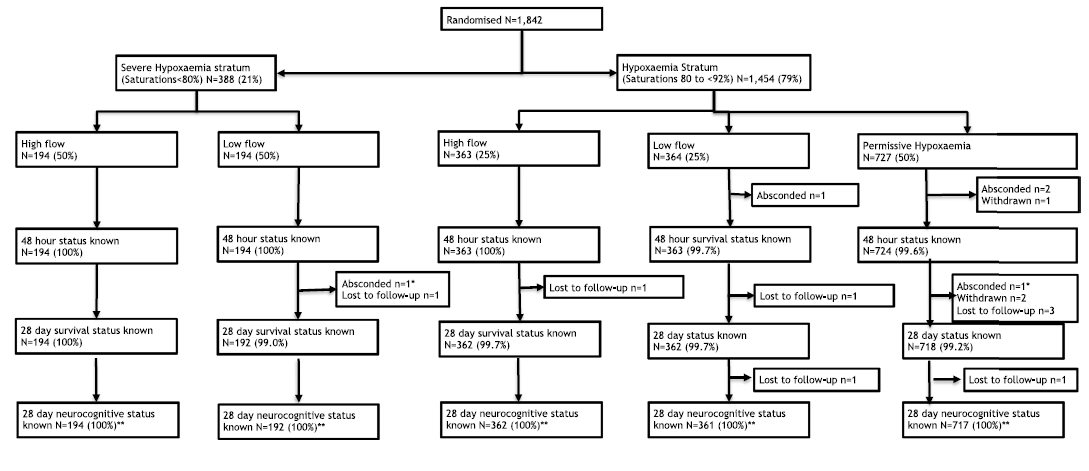


**Fig A:** Flow of patients in COAST (image originally from Maitland et al. (2021)^1^)
* patients were categorised as absconded if they self-discharged from hospital against medical advice and subsequently lost to follow-up before 28 days.
** neurocognitive status was available at 90 days for all children known to have neurocognitive deficit at day 28

**Table A**: Output from the Gamma regression model

| **Variable** | **Incremental cost** | **95% Confidence Interval** | |
| --- | --- | --- | --- |
| *Intervention* | | | |
| Liberal versus permissive | 124.01 | 99.53 | 148.49 |
| HFNT versus LFO | 184.43 | 127.90 | 240.95 |
| *Initial SpO_2_ (grouped)* | | | |
| 70 | -46.55 | -88.74 | -4.35 |
| 80 | -19.94 | -84.46 | 44.57 |
| 90 | -54.00 | -101.82 | -6.18 |
| Intercept | 217.18 | 178.00 | 256.35 |

**References**

1. Maitland K, Kiguli S, Olupot-Olupot P, et al. Randomised controlled trial of oxygen therapy and high-flow nasal therapy in African children with pneumonia. *Intensive Care Med*. 2021;47(5):566-576. doi:10.1007/s00134-021-06385-3

**List of legends**

Fig A: Flow of patients in COAST (image originally from Maitland et al. (2021)^1^)
Table A: Output from the Gamma regression model
